# Quasi-Continuous Wave Pulsed Laser Welding of Copper Lap Joints Using Spatial Beam Oscillation

**DOI:** 10.3390/mi13122092

**Published:** 2022-11-27

**Authors:** Amirhossein Sadeghian, Subhasisa Nath, Yuze Huang, Ranveer S. Matharu, Noppawee Wadee, Nicolas Pembrey, David G. Waugh

**Affiliations:** 1 Institute for Advanced Manufacturing and Engineering, Coventry University, Coventry CV6 5LZ, UK; 2 Advanced Manufacturing Technologies Group, The Welding Institute, Granta Park, Cambridge CB21 6AL, UK

**Keywords:** laser beam welding, copper, spatial beam oscillation, oscillation amplitude, oscillation frequency

## Abstract

Laser beam welding of copper (Cu) using near-infrared radiation is extremely challenging due to its high thermal conductivity and large laser reflectivity. In the present study, the challenges and benefits of using spatial beam oscillation during quasi-continuous wave (QCW) pulsed laser beam welding of 0.4 mm Cu to 1 mm Cu in lap joint configuration are presented. This work demonstrates how laser beam oscillating parameters can be used to control the laser weld quality and laser weld dimensions for Cu-Cu joining. Compared to a non-oscillated laser beam, welds made using laser beam oscillation showed fewer spatters, porosities, and better surface quality. Four levels of oscillating amplitudes (0.2 mm, 0.4 mm, 0.6 mm, and 0.8 mm) and oscillating frequencies (100 Hz, 200 Hz, 300 Hz, and 400 Hz) were compared to reveal the effect of beam oscillation parameters. The weld width was mainly controlled by oscillating amplitude, while weld penetration was affected by both oscillating amplitude and frequency. As the oscillating amplitude increased, the weld width increased while the weld penetration decreased. Increasing the oscillating frequency reduced the weld penetration but had a negligible effect on the weld width. The maximum tensile force of approximately 1944 N was achieved for the joint with a high width-to-depth ratio with an oscillating amplitude of 0.8 mm and an oscillating frequency of 200 Hz.

## 1. Introduction

Due to rising concerns over climate change, automotive manufacturers are shifting their focus from fossil-fuel-powered vehicles to electric vehicles (EVs) [1]. The required energy in this type of vehicle comes from stored electricity in a battery [2]. The EV powertrain typically contains a few hundred to several thousand battery cells connected in series and parallel in the form of a battery pack [3]. In a pouch cell-based battery pack, copper (Cu) and aluminium (Al) are mainly used as positive and negative tabs due to their high electrical conductivity. These tabs are connected to the busbar (current collector), which is also made of Al or Cu, to produce tab-to-busbar joints. As a result, the tab-to-busbar joint includes both dissimilar (Al-Cu, or Cu-Al) and similar (Al-Al, or Cu-Cu) combinations [4,5]. In EV battery manufacturing, there are several joining requirements; (a) high joint strength and toughness; (b) low electrical resistance of the joints, and (c) low-temperature rise during the joining process [6]. At present, resistance spot welding, ultrasonic welding, and laser beam welding are the most widely used techniques for battery cell joining [7]. 

Laser welding has been identified as a promising battery cell joining technique due to its automation capability, fast processing time, and high concentration of energy [8]. However, the use of copper as tab and busbar material makes the laser welding process challenging due to the high thermal conductivity of copper (~390 W/(m.K)) and its poor absorptivity of infrared (IR) laser radiation (around 5% at room temperature). This necessitates applying high-energy inputs to overcome these processing issues [9]. Problems with large amounts of porosities due to the dissolution of oxygen and spatters in the weld region, on account of process instabilities, also exist, which further undermines the process efficiency of laser beam welding [10]. 

Researchers have developed various laser beam welding techniques to improve the weldability of copper. Dadras et al. [11] suggested preheating the copper as a method to amplify the laser absorption and improve the weld quality. Petring et al. [12] studied the effect of laser power, laser intensity, and material properties on the weld geometry; based on that, they suggested optimum process parameters for laser beam micro welding of copper. A combination of two laser beam sources of 515 nm and 1030 nm wavelengths was used by Hess et al. [13]. Implementation of this dual beam method drastically decreased the number of melt ejections while the penetration depth increased, compared to only using a single 1030 nm wavelength laser. Reisgen et al. [14] reported that decreasing the ambient pressure by using laser beam welding in a vacuum can increase the process stability. Chen et al. [15] developed a nano-composite material as an absorber for laser welding of pure copper, which significantly increased the welding efficiency. The possibility of using surface structures to increase the efficiency in the laser welding of copper was investigated by Helm et al. [16]. They reported that processing welding surfaces with ultrashort pulsed (USP) lasers could reduce the reflectance during bead-on-plate laser beam welding. Maina et al. [17] proposed optimising the surface texture to improve light absorption and stabilise the laser beam welding process. Optical coherence tomography (OCT) was used by Will et al. [18] to measure the weld depth and control the quality of laser beam welding of Cu-Cu sheets. A feature called absolute energy was defined to classify different weld statuses. Zhang et al. [19] were able to produce copper joints with a high welding speed of 15 m/min using pressure-assisted laser welding. Electron backscatter diffraction (EBSD) showed that the microstructure of the welds were influenced by both the thermal effect of laser and roller-induced pressure.

A relatively new and cost-effective approach for laser beam welding of highly reflective materials is the use of spatial beam oscillation [20]. Beam oscillation or wobbling increases the temperature of the material leading to higher absorptivity and energy efficiency. The thermal gradient can also be better controlled via wobbling by implementing multiple passages of the laser beam over a single point of the weld, improving weld stability, and reducing spatter and porosity [21]. Some research has been carried out on dissimilar Al-to-Cu and Cu-to-Al welds [22,23], whilst available information on wobble laser beam welding of copper to copper tab-to-busbar joint is more limited. Franco et al. [24] analysed laser beam welding of copper using spatial beam oscillation. They reported the positive impact of beam oscillation on welds due to changes in welding mode, from keyhole mode to conduction mode. Kumar et al. [25] studied the laser beam welding of similar and dissimilar tab-to-busbar joints, including copper-to-copper. Good quality joints without any crack formation were observed due to low-stress generation during laser beam welding. Hummel et al. [26] used spatial and temporal power modulation to control the weld penetration in laser beam welding of copper. They concluded that the position of minimum and maximum laser power is critical for the adjustment of weld seam geometry.

The wobble technique is not just limited to continuous wave (CW) lasers, and benefits can also be realised when applied to pulsed lasers such as millisecond quasi-continuous (QCW) fibre lasers [27]. Therefore, in this paper, a QCW laser with a circular spatial beam modulation was used to weld 0.4 mm Cu (tab) to 1 mm Cu (busbar). The influence of wobbling parameters (oscillation amplitude and frequency) on the weld quality (surface roughness, microstructure, and penetration width and depth) and mechanical strength is investigated. 

## 2. Materials and Methods

### 2.1. Materials

In the present study, a commercially pure (99.9%) high-conductivity grade of copper (C101) was used. C101 electrolytic tough pitch is the normal grade for general electrical applications. The small coupons of 85 mm × 35 mm × 0.4 mm (tab) and 85 mm × 35 mm × 1 mm (busbar) were cut from the large sheets, followed by ultrasonic cleaning in acetone for 15 min. The cleaned coupons were then used for the laser beam welding experiments. Table 1 presents the most relevant physical properties of C101 copper.

### 2.2. Wobble Laser Beam Welding

**Figure 1:**
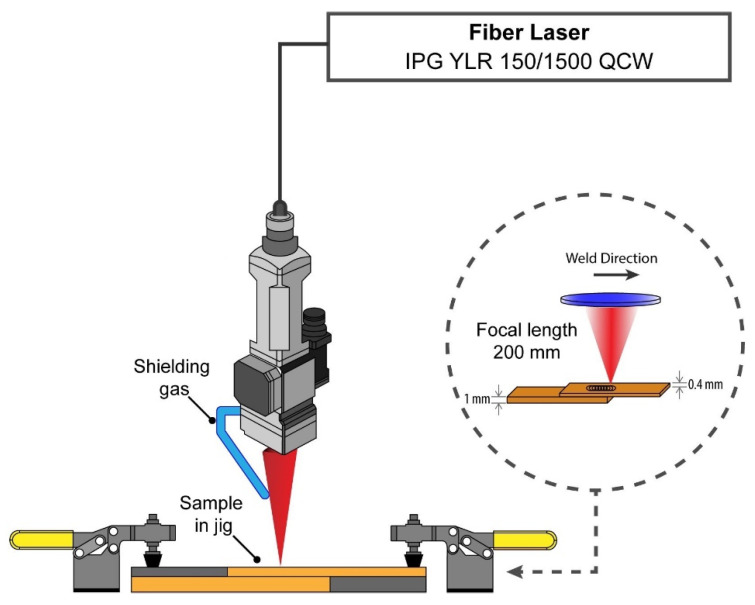
Schematic view of the laser beam welding set-up.

Laser beam welding experiments were conducted using an IPG Photonics fibre laser (YLR 150/1500 QCW). The laser welding was carried out in the pulsed mode with an available maximum peak power of 1500 W. A schematic of the laser welding set-up used in this study is illustrated in Figure 1. A peak power of 750 W was used for laser beam welding. The oscillating frequency and amplitude varied between 100 Hz to 400 Hz and 0.2 mm to 0.8 mm, respectively. All laser welding experiments were conducted with the laser beam focused on the top surface of the top copper sheet (Z = 0). The pulse repetition rate (PRR) was 20 Hz, and the pulse width (PW) was 10 ms in all experiments. The selection of PRR and PW was influenced by the duty cycle limitation of the laser so that the maximum average power of 150 W could be achieved. After initial trials, the linear welding speed (*V_w_*) was fixed at 2 mm/s. A specialist jig was designed to mechanically hold the samples during the laser beam welding experiments. Argon was used as the shielding gas, and the flow rate was 15 L/min. The parameters used for laser beam welding are presented in Table 2.

**Figure 2:**
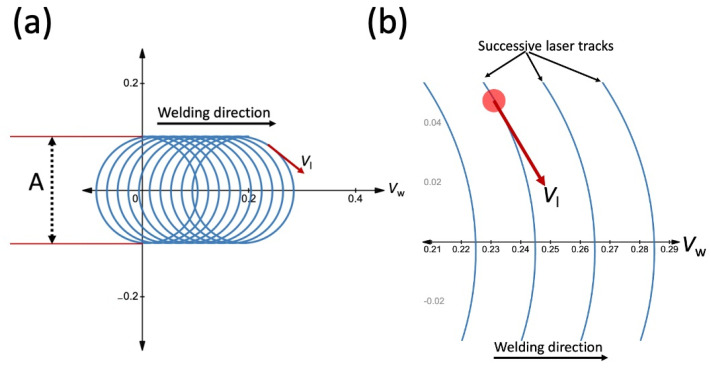
(**a**) Schematic of laser beam oscillation welding (**b**) linear welding speed (*V*_w_) and laser velocity (*V*_l_).

Spatial power modulation of the laser beam was achieved by the superposition of a circular laser beam motion and linear welding, as shown in Figure 2. The motion of the laser beam in a circular path can be expressed as a movement in the *X* and *Y* directions according to Equations (1) and (2), respectively [22].

(1) $$X\;\left(t\right)=V_{w}\;t+\frac{A}{2}\cos\left(\frac{2v_{l}}{A}t\right)\;$$

(2) $$Y\;\left(t\right)=\frac{A}{2}\sin\left(\frac{2v_{l}}{A}t\right)$$

where *V_w_, V_l_*, *t*, *A,* and *f* are the linear welding speed (mm/s), laser velocity (mm/s), time (s), oscillation amplitude (mm), and oscillation frequency (Hz), respectively. 

The superimposed oscillation movement leads to an overlap that can be calculated with Equation (3) for CW lasers. The velocity of the laser at any given point on the circle is related to the oscillation frequency and amplitude according to Equation (4) [22].

(3) $$Overlap\;\left(\%\right)=\left(1-\frac{V_{w}}{fA}\right)\times 100$$

(4) $$V_{l}=\pi fA$$

The interaction time of the laser with the material can be calculated by Equation (5) [29].

(5) $$t_{i}=d_{laser}/V_{l}$$

where *t_i_* is the interaction time, and *d_laser_* is the laser spot size.

Overlap factor, laser velocity, and interaction time were calculated for each wobbling condition based on Equations (3)–(5), which are presented in Table 3. It should be noted that the values calculated for the overlap factor were based on the equation developed for the CW lasers. Due to the pulsed nature of the QCW lasers, the actual wobbling overlap is not consistent throughout the weld line. However, the defined overlap factor is still useful for studying the effect of oscillation amplitude and frequency on wobbling overlap.

### 2.3. Characterisation Techniques

**Figure 3:**
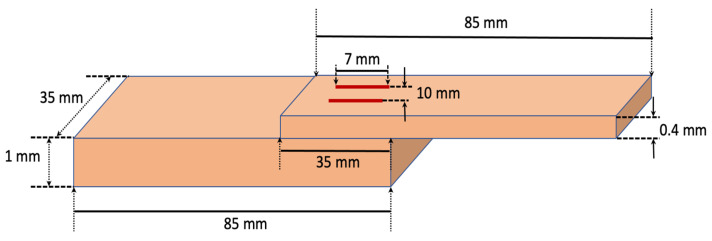
Schematic arrangement of coupons in a lap-joint configuration for tensile lap shear tests.

A stereo microscope (S6D, Leica, UK) and a focus variation microscope (InfiniteFocus G5, Bruker Alicona, Austria) were used to probe the process parameter effect on the weld surface quality. For weld surface analysis, a 10 mm weld length was used. Aerial topography measurements of surface texture were performed using a 20× magnification objective lens (NA 0.4; X, Y Lateral measurement range 0.81 mm), with an estimated vertical resolution of 0.052 μm and a lateral resolution of 2.936 μm. This was sufficient enough to capture measurable features across the weld surfaces, where the minimum measurable roughness, Ra, with the lens was 0.15 μm. For weld cross-sectional microstructure analysis, the samples were cut using electrical discharge machining (EDM) and then ground using 400, 600, 800, and 1200 SiC papers. This was followed by etching in 100 mL Ethanol + 25 mL Hydrochloric acid + 5 mL Ferric chloride. The weld cross-sections were analysed using a Leica DFC295 optical microscopy to understand weld microstructure, geometrical features, and the size of grains. The microhardness profile in the weld samples was measured using a Vickers microhardness tester with a load of 50 gf with a dwell time of 10 s. Overall, a total of 15 indents were used. The tensile shears tests were carried out with a crosshead speed of 1 mm/min using a universal tensile machine (INSTRON) at room temperature. Two weld lines with individual weld lengths of 7 mm were produced parallel to the longitudinal direction of the coupon. The schematic of the coupons used for the tensile shear tests is shown in Figure 3. The maximum shear load was measured from the load–displacement curve. A total of three tests were performed to calculate the mean values. The failed weld coupons were analysed to understand the mechanism of failure.

## 3. Results and Discussion

### 3.1. Surface Quality of Welds

**Figure 4:**
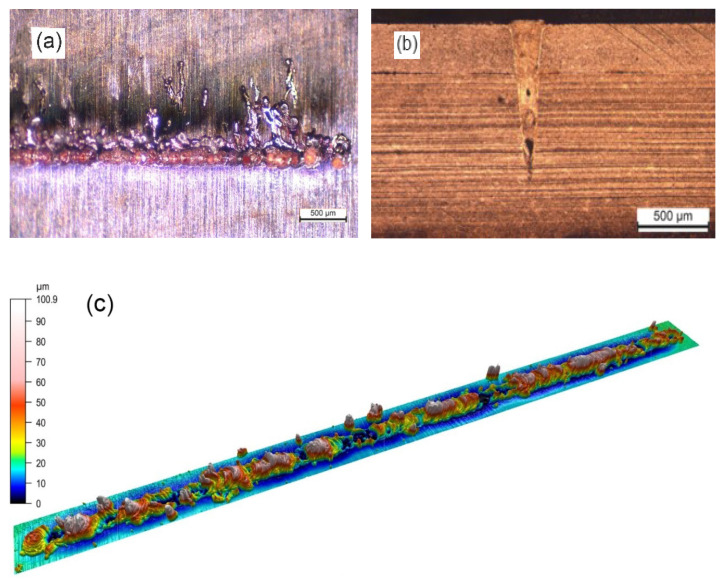
(**a**) Top view, (**b**) cross-section, and (**c**) 3D surface plot of the sample made without beam oscillation. Laser beam welding parameters were P = 750 W, PRR = 20 Hz, PW = 10 ms, V_w_ = 2 mm/s, and gas flow = 15 L/min.

Figure 4 shows the top surface, cross section and 3D surface plot of the copper-to-copper tab-to-busbar joint made without beam oscillation. A poor surface quality with a large number of melt ejections and surface voids is observed due to process instability caused by the low laser absorption rate of copper. The presence of large pores due to keyhole rupture and low weld width is evident from the weld cross-section. Furthermore, the 3D plot shows the uneven and rough surface of the weld. The low quality of the non-wobbling joint leads to reduced mechanical strength and electrical conductivity. 

**Figure 5:**
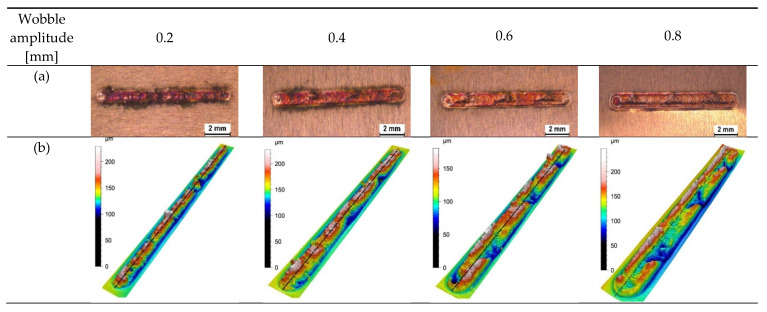

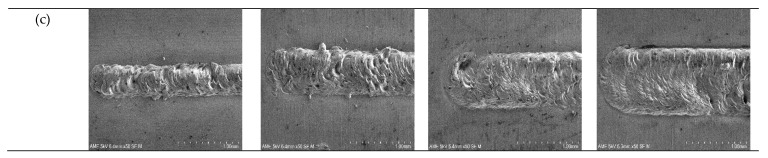
Top view of samples produced with different wobbling amplitudes (0.2 mm, 0.4 mm, 0.6 mm, and 0.8 mm) using (**a**) optical microscopy, (**b**) 3D surface plot, and (**c**) scanning electron microscopy (P = 750 W, PRR = 20 Hz, PW = 10 ms, F = 200 Hz, V_w_ = 2 mm/s, and gas flow = 15 L/min).

**Figure 6:**
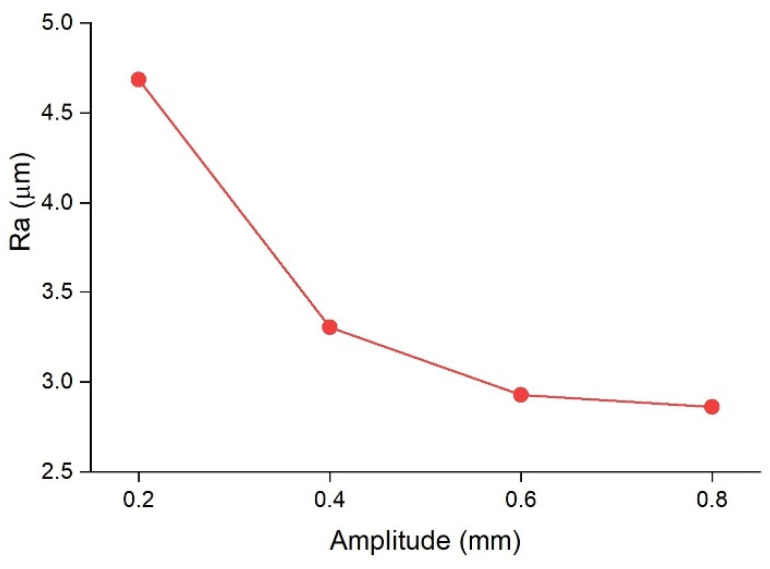
Roughness value (R_a_) obtained for samples with 0.2 mm, 0.4 mm, 0.6 mm, and 0.8 mm oscillating amplitudes along the centre line of the welds. The oscillating frequency was kept constant at 200 Hz.

Figure 5 presents the top view of the welds made with an oscillation frequency of 200 Hz and an oscillation amplitude of 0.2 mm, 0.4 mm, 0.6 mm, and 0.8 mm using optical microscopy (OM), 3D surface plotting, and scanning electron microscopy (SEM). The surface roughness (R_a_) along the weld line in the middle of the welds is also presented in Figure 6. An unstable process, which is indicated by a high R_a_ value, is caused by a weld with considerable spatters and melt ejections. Compared to laser welding under non-wobbling conditions, the weld surface is better formed; it is more uniform and with less surface roughness. Moreover, drastically fewer surface voids and spatters can be observed in samples with beam oscillation. As oscillation amplitude increased, fewer spatters and voids were created as the sample with the 0.8 mm amplitude showed the smoothest surface with the lowest roughness value of just below 3 µn. Therefore, it can be hypothesised that the melt pool ejections, which create spatters and also lead to surface voids, can be effectively suppressed by higher oscillation amplitudes.

**Figure 7:**
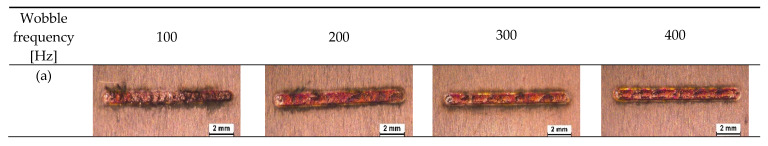

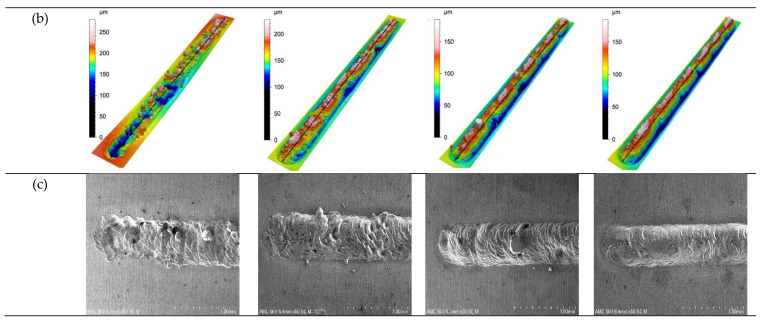
Top view of samples produced with different wobbling frequencies (100 Hz, 200 Hz, 300 Hz, and 400 Hz) using (**a**) optical microscopy, (**b**) 3D surface plotting, and (**c**) scanning electron microscopy (P = 750 W, PRR = 20 Hz, PW = 10 ms, A = 0.4 mm, V_w_ = 2 mm/s, and gas flow = 15 L/min).

**Figure 8:**
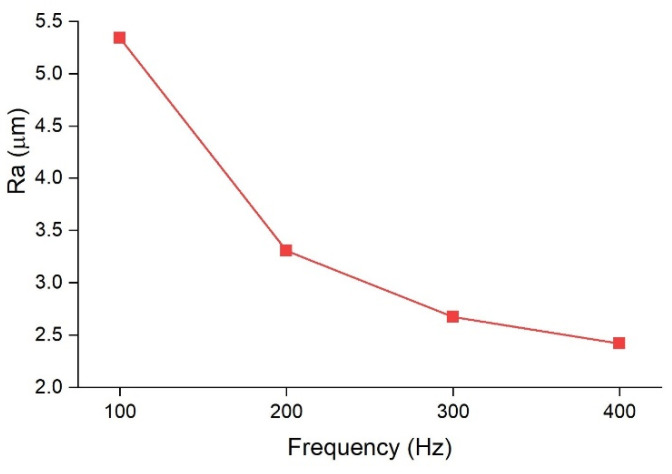
Roughness value (R_a_) obtained along the centre line of the welds for 100 Hz, 200 Hz, 300 Hz, and 400 Hz oscillating frequencies. The oscillating amplitude was kept constant at 0.4 mm.

The effect of oscillation frequency on the surface quality of copper-to-copper joints was also investigated. Samples with a constant amplitude of 0.4 mm and varying oscillation frequencies of 100 Hz, 200 Hz, 300 Hz, and 400 Hz were compared. The top views of the welds using OM, 3D surface plotting, and SEM are presented in Figure 7. Figure 8 also shows the surface roughness along the weld line in the middle of the welds. As can be seen from Figure 7, increasing the oscillation frequency improved the surface uniformity by creating smoother ripples and reduced the number of surface voids and spatters. The roughness value also decreased with the increase in oscillation frequency to around 2.5 µm for the sample produced with the oscillation frequency of 400 Hz. 

The main reason for the decrease in surface roughness for higher oscillation amplitudes and frequencies could be justified by the overlap factor. Based on Eq. 3, the overlap factor has a direct relationship with the oscillation frequency and amplitude. Higher overlap means wobbling circles are closer to each other; thus, a fine and smoother surface is formed.

### 3.2. Weld Cross-Section

**Figure 9:**
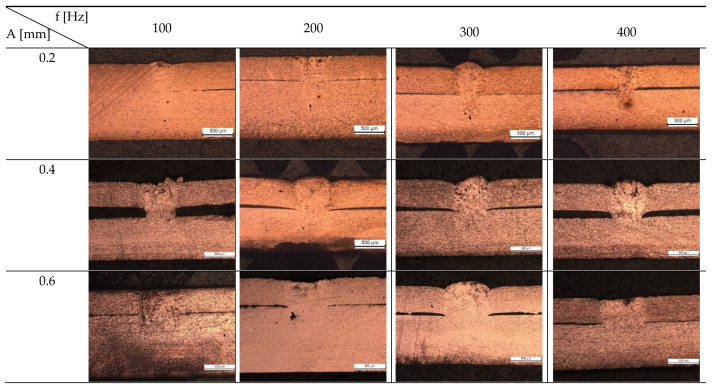

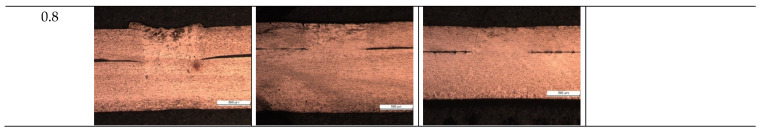
Cross-section of the welds with different oscillating amplitudes (0.2 mm, 0.4 mm, 0.6 mm, and 0.8 mm) and oscillation frequencies (100 Hz, 200 Hz, 300 Hz, and 400 Hz) (A: oscillating amplitude; f: oscillating frequency).

**Figure 10:**
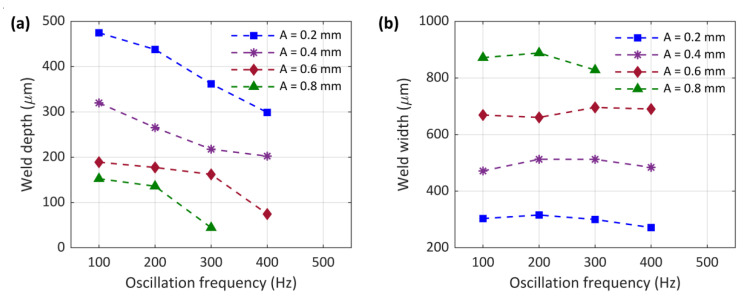
(**a**) Weld depth and (**b**) weld width versus oscillating amplitudes (0.2 mm, 0.4 mm, 0.6 mm, and 0.8 mm) and oscillation frequencies (100 Hz, 200 Hz, 300 Hz, and 400 Hz).

The cross-section of the welds with varying oscillation amplitudes (0.2 mm, 0.4 mm, 0.6 mm, and 0.8 mm) and frequencies (100 Hz, 200 Hz, 300 Hz, and 400 Hz) are presented in Figure 9. Furthermore, Figure 10 illustrates the weld depth and interface width variation with different oscillating amplitudes and frequencies. Compared to the sample without laser beam wobbling, the penetration depth is generally smaller, while the interface width is significantly increased. No crack can be seen in samples made with circular wobbling. Moreover, less porosity is generally observed in these samples compared to conventional laser beam welding, although samples with low wobbling amplitude (0.2 mm) showed a small number of porosities, which improved in higher amplitudes. As the oscillation amplitude increased, the welding mode changed from keyhole to conduction, inhibiting the formation of keyhole-related porosities. Undercut was also formed in some of the samples with oscillation amplitudes of 0.2 mm and 0.4 mm due to spattering.

For samples made with laser beam oscillation, the penetration depth was controlled by both the oscillation amplitude and frequency, whereas the change in weld width was mainly driven by oscillation amplitude. As the oscillation amplitude increased, the penetration depth decreased. A similar trend was identified for oscillation frequency. Although based on calculations presented in Table 3, increasing the oscillation amplitude and frequency both lead to a higher overlap factor, the laser velocity was also affected and increased, which reduced the laser–material interaction time. It can be concluded that the interaction time was the dominating factor determining the weld penetration depth and the increase in overlap factor was not enough to compensate for the reduction in laser–material interaction time. When the laser–material interaction time was low due to high oscillation amplitude and frequency, less energy was deposited into the material, leading to lower heat input and, thus, a smaller scale of the melt pool and penetration depth. As the oscillation amplitude increased, a noticeably lower penetration depth was induced under the low interaction time, which even caused no fusion at the interface of the sample with the highest oscillation frequency of 400 Hz. 

A higher oscillation amplitude means a larger laser–material interaction area. It should be noted that higher oscillation amplitude gave rise to lower laser–material interaction time due to higher laser velocity, but the laser interacting with a larger area as the oscillation amplitude increased. Therefore, by increasing the wobble amplitude, the weld width significantly increased. For example, changing the oscillation amplitude from 0.2 mm to 0.8 mm increased the interface width from 300 µm to 870 µm for the sample with an oscillation frequency of 100 Hz. Due to the increase in weld width and decrease in weld penetration, the shape of the weld cross section almost turned rectangular. Contrary to oscillation amplitude, the oscillation frequency had a negligible effect on the interface width. For example, increasing the oscillation frequency from 100 Hz to 400 Hz in a constant oscillation amplitude of 0.4 mm only increased the interface width from 471 µm to 484 µm. Therefore, it can be hypothesised that the interface width was mainly governed by the oscillation amplitude.

### 3.3. Weld Microstructure

**Figure 11:**
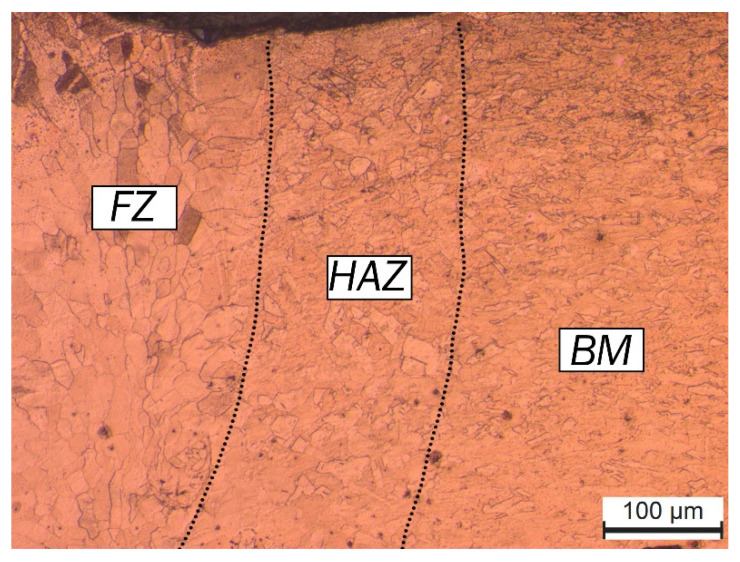
Optical micrograph of the weld region in top sheet of copper-to-copper joint made with an oscillation amplitude of 0.2 mm and an oscillation frequency of 100 Hz showing FZ, HAZ, and BM.

Figure 11 shows the optical micrograph of the sample produced with an oscillation amplitude of 0.2 mm and an oscillation frequency of 100 Hz. Three distinct microstructural zones can be defined from this figure; the fusion zone (FZ), the adjacent heat-affected zone (HAZ), and the base metal (BM). The BM is the region in which the heat was not sufficient to create any microstructural changes. The initial microstructure in this region, which consisted of the fine equiaxed grains, remained unaffected. In the HAZ, the heat input was not enough to melt the copper but caused recrystallisation and grain growth due to overheating, creating an overgrown equiaxial microstructure. In the FZ, the region where the material was melted, mostly coarse columnar grains can be observed. Pure copper does not undergo a solid-state transformation, and it can be seen from Figure 11 that the main difference in BM and HZ microstructure was the difference in the size of the grains. However, the structure of grains in the FZ was also altered.

### 3.4. Mechanical Properties

**Figure 12:**
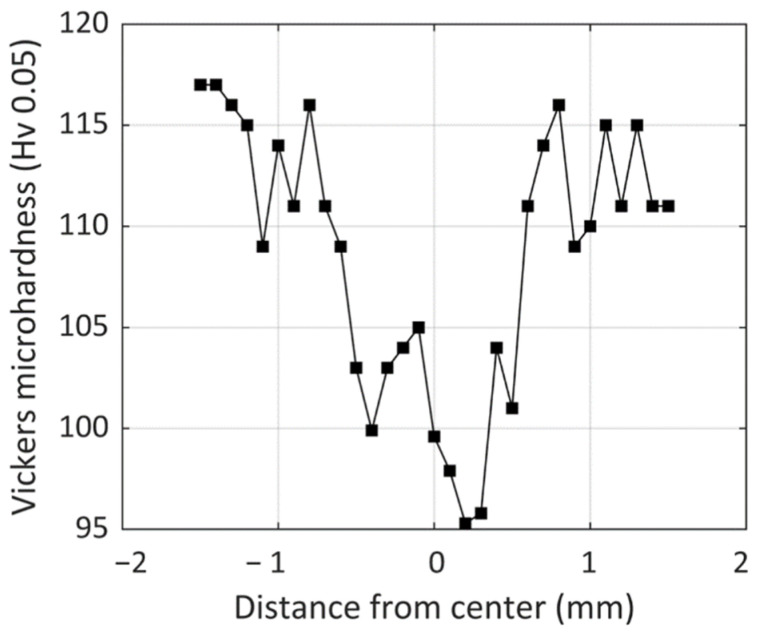
Microhardness variation across the weld (BM, HAZ, and FZ regions) for the sample with an oscillation amplitude of 0.6 mm and oscillation frequency of 300 Hz.

Microhardness variation across the weld in the sample, with an oscillation amplitude of 0.6 mm and an oscillation frequency of 300 Hz, is presented in Figure 12. As can be seen, there is a decrease in microhardness by moving along from the BM to the HAZ and then FZ, with FZ having the lowest microhardness value of around 95 Hv. According to the Hall–Petch effect, the hardness decreases with the grain size increase; this softened zone corresponds with the grain coarsening in the HAZ and FZ in contrast to smaller grains of BM.

**Figure 13:**
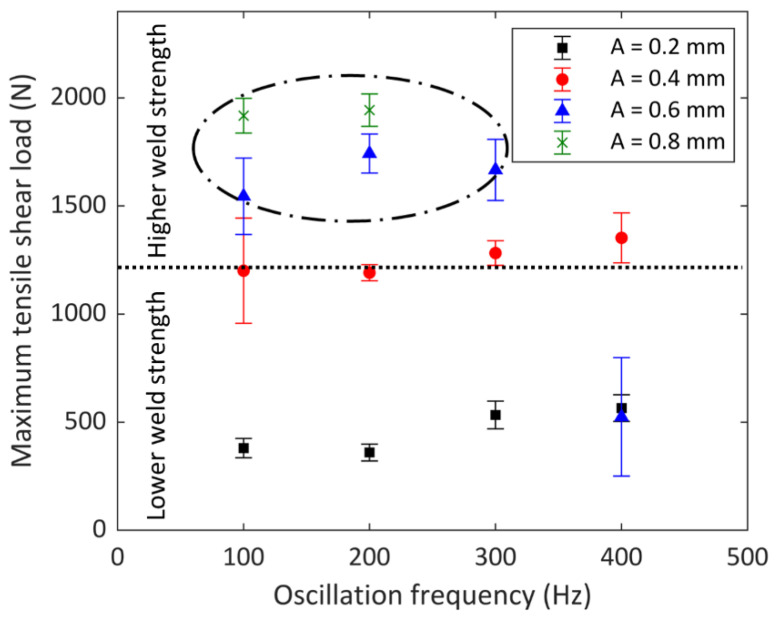
Variation in maximum tensile shear force versus oscillation parameters (laser peak power = 750 W, PRR = 20 Hz, PW = 10 ms, V_w_ = 2 mm/s).

Figure 13 illustrates the maximum tensile shear strength of copper-to-copper joints for samples with different oscillation amplitude and oscillation frequencies. The tensile shear test samples had two parallel longitudinal weld lines. The weld bead geometry, meaning the interface width and the penetration depth, are the key factors determining joint mechanical strength. Insufficient penetration depth and small weld interface reduce weld strength drastically. As can be seen from Figure 13, oscillation amplitude has a direct effect on joint strength. Higher oscillation amplitudes (0.6 mm and 0.8 mm) led to a higher contact area (interface width) between the two sheets, which resulted in higher mechanical strength in joints with sufficient penetration depths. The optimum tensile shear strength was identified at an oscillation amplitude of 0.6 mm; oscillation frequencies of 100 Hz, 200 Hz, and 300 Hz; and at a wobble amplitude of 0.8 mm for 100 Hz and 200 Hz oscillation frequency. The maximum tensile shear load of around 1944 N was achieved for the sample with an oscillation amplitude of 0.8 mm and an oscillation frequency of 200 Hz. It seems that changing the oscillation frequency at constant amplitude had a slight influence on the joint strength due to its negligible effect on weld width. However, due to the effect of oscillation frequency on penetration depth, in samples with high oscillation amplitudes (0.6 mm and 0.8 mm), the penetration depth fell below 100 µm with high oscillation frequencies resulting in ‘under-weld’ or lack of fusion defect. The lack of fusion was observed in 0.6 mm and 0.8 mm welds with an oscillation frequency of 400 Hz and in the 0.8 mm sample with oscillation frequencies of 300 Hz and 400 Hz. In those samples, the maximum tensile shear load was either drastically reduced, or the samples fell apart before testing. As a result, when sufficient penetration depth was retained, the joint strength was governed by the weld width. Thus, adjusting both oscillation amplitude and frequency was the key to controlling the mechanical strength of welds.

## 4. Conclusions

In this study, the effect of oscillation amplitude and frequency on the surface quality, weld geometry, and mechanical strength of copper-to-copper tab-to-busbar joints made with quasi-continuous wave (QCW) pulsed laser was investigated. The findings can be summarised below:

1. Laser welding with spatial beam oscillation greatly influenced surface roughness, weld geometry, and mechanical strength compared to joints made without beam oscillation. A smoother surface with fewer surface voids, porosities, and cracks was observed in the samples with beam wobbling, especially in joints with higher oscillation amplitudes (0.6 mm and 0.8 mm).
2. The width-to-depth ratio of welds was mainly controlled by oscillation amplitude. As the oscillation amplitude increased, the weld width increased, and the weld penetration depth decreased. The oscillation frequency did not have a significant effect on the weld width but increasing the oscillation frequency reduced the penetration depth, causing a lack of fusion in samples with high amplitude.
3. A complex microstructure was observed in the weld region of joints made with beam oscillation with columnar grains in the FZ and coarser grains in the HAZ compared to BM. The microhardness profile across the joint showed a decrease in microhardness values from BM to HAZ and FZ due to grain coarsening.
4. The mechanical strength of welds was mainly governed by the weld width as long as the penetration depth was sufficient (over 100 µm). The tensile shear load significantly increased with the increase in weld width. A range for oscillation amplitude and oscillation frequency (100 Hz and 200 Hz for the 0.8 mm sample and 100 Hz, 200 Hz, and 300 Hz for the 0.6 mm sample) was defined, corresponding to the highest tensile shear loads.

## Figures and Tables

**Table 1:** Physical properties of C101 copper [28].

Material	C101
Melting point [°C]	1083
Density [g/cm^3^]	8.92
Electrical resistivity [×10^−6^ Ω·m]	0.0171
Thermal conductivity [W/m·k]	391.1
Thermal expansion [×10^−6^ K^−1^]	16.9

**Table 2:** Laser beam welding parameters.

Parameter	Value/Type
Laser spot size [µm]	28
Focus position	On top surface
Peak power [W]	750
Pulse repetition rate [Hz]	20
Pulse width [ms]	10
linear welding speed [mm/s]	2
Oscillation amplitude [mm]	0.2–0.8
Oscillation frequency [Hz]	100–400
Shielding gas type	Argon
Gas flow rate [l/mm]	15

**Table 3:** The overlap factor, laser velocity, and interaction time for different oscillation amplitudes and frequencies.

Sample No.	Oscillation Amplitude [mm]	Oscillation Frequency [Hz]	Overlap Factor [%]	Laser Velocity [mm/s]	Interaction Time [ms]
1	0.2	100	90	62.8	0.445
2	0.2	200	95	125.6	0.222
3	0.2	300	96.7	188.4	0.148
4	0.2	400	97.5	251.2	0.111
5	0.4	100	95	125.6	0.222
6	0.4	200	97.5	251.2	0.111
7	0.4	300	98.3	376.8	0.074
8	0.4	400	98.75	502.4	0.055
9	0.6	100	96.7	188.4	0.148
10	0.6	200	98.3	376.8	0.074
11	0.6	300	98.9	565.2	0.049
12	0.6	400	99.2	753.6	0.037
13	0.8	100	97.5	251.2	0.111
14	0.8	200	98.75	502.4	0.055
15	0.8	300	99.2	753.6	0.037
16	0.8	400	99.4	1004.8	0.027

## Data Availability

All data generated in this study can be found within the paper.
